# SPServer: split-statistical potentials for the analysis of protein structures and protein–protein interactions

**DOI:** 10.1186/s12859-020-03770-5

**Published:** 2021-01-06

**Authors:** Joaquim Aguirre-Plans, Alberto Meseguer, Ruben Molina-Fernandez, Manuel Alejandro Marín-López, Gaurav Jumde, Kevin Casanova, Jaume Bonet, Oriol Fornes, Narcis Fernandez-Fuentes, Baldo Oliva

**Affiliations:** 1 Structural Bioinformatics Lab, Department of Experimental and Health Science, Universitat Pompeu Fabra, 08003 Barcelona, Catalonia Spain; 2 Laboratory of Protein Design and Immuno-Enginneering, School of Engineering, Ecole Polytechnique Federale de Lausanne, 1015 Lausanne, Vaud Switzerland; 3 Centre for Molecular Medicine and Therapeutics, Department of Medical Genetics, BC Children’s Hospital Research Institute, University of British Columbia, Vancouver, BC V5Z 4H4 Canada; 4 Department of Biosciences, U Science Tech, Universitat de Vic-Universitat Central de Catalunya, Vic 08500 Barcelona, Catalonia Spain; 5 Institute of Biological, Environ-Mental and Rural Sciences, Aberystwyth University, Aberystwyth, SY23 3EB UK

**Keywords:** Protein structure evaluation, Protein structure quality assessment, Protein structure prediction, Protein–protein interaction, Protein–protein evaluation, Knowledge-based potential

## Abstract

**Background:**

Statistical potentials, also named knowledge-based potentials, are scoring functions derived from empirical data that can be used to evaluate the quality of protein folds and protein–protein interaction (PPI) structures. In previous works we decomposed the statistical potentials in different terms, named Split-Statistical Potentials, accounting for the type of amino acid pairs, their hydrophobicity, solvent accessibility and type of secondary structure. These potentials have been successfully used to identify near-native structures in protein structure prediction, rank protein docking poses, and predict PPI binding affinities.

**Results:**

Here, we present the SPServer, a web server that applies the Split-Statistical Potentials to analyze protein folds and protein interfaces. SPServer provides global scores as well as residue/residue-pair profiles presented as score plots and maps. This level of detail allows users to: (1) identify potentially problematic regions on protein structures; (2) identify disrupting amino acid pairs in protein interfaces; and (3) compare and analyze the quality of tertiary and quaternary structural models.

**Conclusions:**

While there are many web servers that provide scoring functions to assess the quality of either protein folds or PPI structures, SPServer integrates both aspects in a unique easy-to-use web server. Moreover, the server permits to locally assess the quality of the structures and interfaces at a residue level and provides tools to compare the local assessment between structures.

**Server address:**

https://sbi.upf.edu/spserver/.

## Background

Three-dimensional (3D) structures of proteins and protein–protein interactions (PPIs) are essential to understand most biochemical functions of cells and living organisms. Yet, the amount of experimentally determined 3D structures is limited, especially for protein complexes. Structural models derived by computational methods can be used to close the gap between the number of sequences and structures. In the recent CASP13 competition, we have observed a dramatic progress in the quality of the template-free models made by novel computational methods involving deep learning techniques [1]. However, these methods need to be complemented by evaluation methods to know the margins of accuracy when we study the role of structural models in a biological system [2].

Evaluation methods can be classified into two categories: single- and multiple-model methods. Single-model methods only require one model as input, whereas multiple-model methods require several. The latter ones take advantage of the similarity between the distinct models to evaluate them, but they are not based on the properties of the model itself. In contrast, single-model methods are often based on the geometric and energetic analysis of the model coordinates, although some of them may also use additional information (e.g. for evolutionary related proteins) [3, 4].

For single-model methods, the most common approach is to use knowledge-based potentials, i.e. scoring functions derived from the analysis of empirical data [5]. Several computational methods have been implemented from knowledge-based potentials [6–8]. Split-Statistical Potentials (SPs) are knowledge-based potentials that consider the frequency of pairs of residues in contact and include their structural environment, such as solvent accessibility and type of secondary structure. Previously, we demonstrated that SPs can be used to: (1) identify near-native protein decoys in structure prediction [9]; and (2) rank protein–protein docking poses [10, 11]. SPs compared favorably against 115 scoring functions on a docking decoy benchmark [12] and were successful at predicting binding energies of PPIs without requiring the native structures of the complexes [13].

Many scoring methods have been proposed to assess the quality of protein fold models [6–8, 14–18]. However, very few can be easily accessed as web servers by the non-specialized user. In most cases, the web servers have a reduced input flexibility (i.e. only accept models in PDB format, require chain identifiers and protein sequences, or do not accept multiple structures) and a complicated visualization of the results (i.e. do not permit to download results or do not have 3D visualization capabilities).

Here, we present the Split-Statistical Potentials Server (SPServer) featuring our SPs for the evaluation of protein structures and PPIs. The web server has been designed to facilitate its use and the interpretation of results. When analyzing protein folds, the server returns global scores and shows score profiles along the protein sequence to identify potentially problematic regions in the structure. When analyzing PPIs, the server returns global scores and score maps of the interfaces. The SPServer identifies stabilizing and disrupting residue pairs that can be used as starting point for follow up protein engineering.

## Implementation

**Fig. 1:**
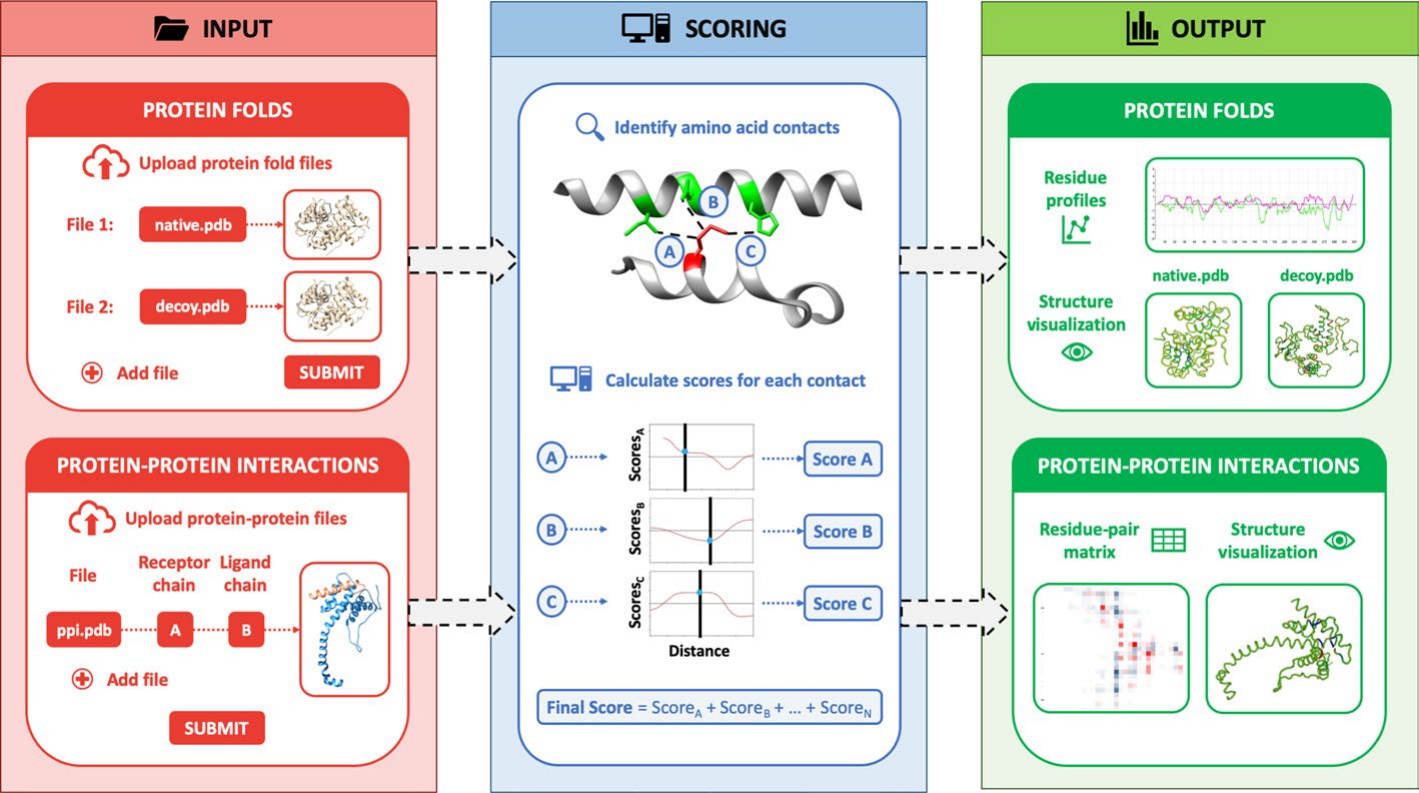
General scheme of the functioning of the SPServer. The web server is divided into three sections: input, to upload either single protein structures (for fold analyses) or binary complexes (for protein–protein interaction analyses); scoring, to score the quality of the single and complex structures; and output, to display the local profiles of single structures and heatmap of residue-residue scores in the interface of the input binary complexes

The overall implementation of the web server is summarized in Fig. 1 and explained in detail as follows:

### Input

As input, users have to provide the structures of one or more proteins or protein complexes. The server input is flexible; users can provide either PDB structures, mmCIF files or compressed directories containing the structures to analyze. Users also have to select the parameter used to define residue contacts (i.e.12 Å cut-off between their β-carbons—option Cβ—or 5 Å between any atom of each residue—option MIN—). Often the structures used as input are produced by modelling or fold prediction approaches, because we are interested in checking the quality of models rather than the quality of experimental structures. In the case of structures of single proteins or folds, the most common methods to produce them are by homology modelling (e.g. by MODELLER [19]), remote homology (e.g. by PHYRE [20] or FUGUE [21]), by threading and ab initio fold prediction (e.g. by I-TASSER [22], THREADER [23], or in particular for sequences in CASP13 using AlphaFold [24]), or protein structure design (e.g. with Rosetta [25]). For protein–protein interactions the structures may be produced by template homology (e.g. from Interactome3D [26], PrePPI [27] or MODPIN [28]), template docking (e.g. by ICM [29]), docking (e.g. by pyDOCK [30], FTDOCK [31], V-D2OCK [32], PatchDock [33] or ZDOCK [34]) or directed docking (e.g. RosettaDock [25] and HADDOCK [35]).

### Scoring

The first step of the scoring process is to identify the contacts between residues from the same protein (to score protein folds) or from different proteins (to score PPIs). These contacts consider the amino acids type, the distance between them, and environmental features such as the type of secondary structure or the degree of exposure of the amino acids. SPs provide a score for each one of these contacts. We obtain the score of a structure by performing the sum of scores of all its contacts. We can also get the scores of individual amino acids by performing the sum of scores of all the contacts of that residue. This can be used to define a score profile along the protein sequence. Residue scores can be averaged using a sliding window of size defined by the user along the protein sequence in order to smooth the profile.

We defined SPs in previous works [9, 10] using the description of a potential of mean force (PMF), say the features describing an amino acid are defined by θ, with: *θ* = *(secondary structure, polar character, degree of exposure)*. Then we define the potentials as in Eqs. 1–5:

1 $${PMF}_{PAIR}\left(a,b\right)=-{k}_{B}T log\left(\frac{P\left(a,b | {d}_{ab}\right)}{P\left(a\right) P\left(b\right) P\left({d}_{ab}\right)}\right)$$

2 $${PMF}_{LOCAL}\left(a,b\right)={k}_{B}T log\left(\frac{P\left(a | {\theta }_{a}\right) P\left({\theta }_{a}\right)}{P\left(a\right)}\right)+{k}_{B}T log\left(\frac{P\left(b | {\theta }_{b}\right) P\left({\theta }_{b}\right)}{P\left(b\right)}\right)$$

3 $${PMF}_{3D}\left(a,b\right)={k}_{B}T log\left(P\left({d}_{ab}\right)\right)$$

4 $${PMF}_{3DC}\left(a,b\right)={k}_{B}T log\left(\frac{P\left({\theta }_{a},{\theta }_{b}\right) | {d}_{ab}}{P\left({\theta }_{a},{\theta }_{b}\right)}\right)$$

5 $${PMF}_{S3DC}\left(a,b\right)={-k}_{B}T log\left(\frac{P\left(a,b | {d}_{ab}, {\theta }_{a},{\theta }_{b}\right) P({\theta }_{a},{\theta }_{b})}{P\left(a,b | {\theta }_{a},{\theta }_{b}\right) P\left({\theta }_{a},{\theta }_{b} | {d}_{ab}\right)}\right)$$

with k_B_ the Boltzmann constant, T the standard temperature (300 K), θ_a_, and θ_b_ the features of amino acids a and b, and d_ab_ the distance between both residues. The terms P(·) denote the probabilities of observing interacting pairs (with or without conditions). For instance, P(a,b|d_ab_) is the conditional probability that residues a,b interact at distance smaller than or equal to d_ab_, and P(d_ab_) is the probability of finding any pair of residues interacting at distance smaller than or equal to d_ab_.

The scores *PAIR, ELOCAL, E3D, E3DC, and ES3DC* are obtained by summing the PMF with the corresponding subindex of each pair of interacting residues a, b, either of the same protein (for fold) or between two interacting proteins (for PPIs), as in Eq. 6:

6 $$E=\sum_{a,b}PMF\left(a,b\right)$$

We proved [9] that the classical statistic potential, PAIR, can be approximated to:

7 $$PAIR=ES3DC-E3DC+E3D-ELOCAL+\varepsilon$$

With a residual ε that accounts for the reference state and becomes noise centered at 0 upon normalization (i.e. when transformed in Z-scores, see further). Hence, given that E3D nullifies when normalizing the scores and ε is irrelevant, we define another score, ECOMB, as:

8 $$ECOMB=ES3DC-E3DC-ELOCAL$$

Furthermore, these potentials can be used to generate a profile per amino acid position along the sequence by summing the energies of the contacts of each residue.

In conclusion, the SPServer has 6 types of SPs available that differ on the environmental features considered for the contact definition: (1) *ES3DC* considers residue frequencies along distances and their environments (i.e. hydrophobicity of each amino acid, solvent accessibility and secondary structure); (2) *E3DC* considers frequencies along distances of pairs referred by the hydrophobicity of the amino acids and the rest of their environments; (3) *PAIR* considers amino acid frequencies along distances; (4) *ELOCAL* considers amino acid frequencies on a particular environment; (5) *E3D* considers the frequencies of any pair of residues along distances; and finally, (6) *ECOMB* combines *ES3DC*, *ELOCAL* and *E3DC* scores [9]. Additionally, Z-scores are provided for each one of these scoring functions by normalizing the scores with respect to the average and standard deviation of 1000 random sequences with the same structure. Similarly, scoring profiles can also be transformed into Z-scoring profiles by normalizing with respect to the 20 possible amino acids in each position. As calculated, scores are proxy measures for energy, and thus, the lowest the score is, the closer the model is to the native-like structure.

### Output for protein folds

For a set of protein folds, the SPServer outputs: (1) the global scores (raw and normalized) of all SPs; and (2) the scoring profile per residue (local scores) along the protein sequence. Global scores account for the overall quality of structural models, while per-residue score plots pinpoint problematic regions of the models that likely have either a wrong conformation or contacts with a wrongly modelled region.

### Output for protein–protein interactions

For PPIs, the server outputs: (1) global scores for the quality of the interface between the two interacting proteins; (2) a measure of the penetration between two proteins to assess for steric clashes at the interface; and (3) interface maps with the scores of residue contact-pairs between the two proteins. Global scores inform on the overall quality of the interaction (i.e. for ranking docking poses). The measurement of steric hindrances is indicated in a color legend depending on the relevance of the clashes (see Additional file 1: Data and Additional file 2: Figure S1 for details). Finally, interface maps allow for detailed exploration of the protein interfaces at residue level. The server also provides different tools to smooth and compare interface maps.

## Results and discussion

### Case study 1: Evaluation of the structural models of Cysteine synthase A

**Fig. 2:**
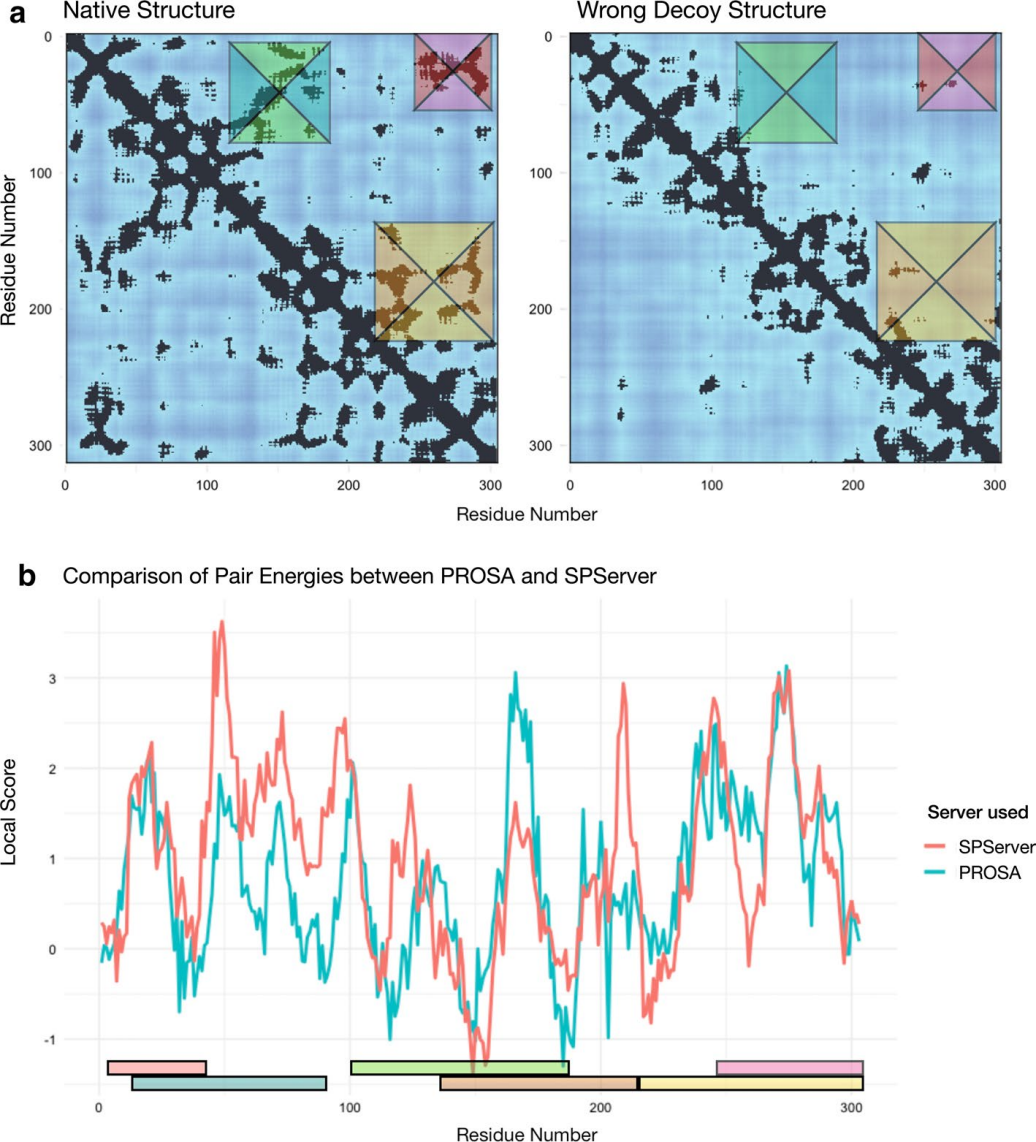
Comparison of the residue pair scores for the native and wrong decoy structures of cysteine synthase calculated with PROSA and SPServer. **a** Residue-residue contact maps are shown at the top, with green/blue, pink/red and brown/yellow colors identifying native contacts that have been lost when comparing the native structure and the wrong decoy, where native contacts are lost. **b** Local profile of the difference between the scores per residue of the native structure and the wrong decoy (in red are shown the scores of PAIR and in blue the scores of Pair potential of PROSA). The regions highlighted in the contact maps are also shown on the X-axis above the residue number, showing a coincidence between high scores and the regions where the wrong decoy differs from the native structure

We compared the native structure of Cysteine synthase A from *E. coli* with two decoys of predicted structures: a near-native structure and a wrong decoy. All structures were retrieved from the CASP12 dataset (codes T0861, T0861TS275_2 and T0861TS321_1) [36]. The global scores rank the native structure with the lowest score, followed by the near-native and the wrong decoy (see Additional file 13: Table S1). Local score profiles of the native and the near-native structures are very similar, while the profile of the wrong decoy is different (see Additional file 3: Figure S2 and Additional file 4: Figure S3). Moreover, we compared the results of SPServer PAIR potential with a standard statistical potential (PROSA [6]). Both potentials show similar differences between the profiles of the native structure and the wrong decoy (Pearson correlation coefficient = 0.50), and highlight the residue-residue contact areas corresponding with wrongly modelled regions of the decoy structure (see Fig. 2).

### Case study 2: Mutation in the interaction between BAX and BID

The interaction of BAX with BID mediates the insertion of BAX in the outer mitochondrial membrane, which induces apoptosis [37]. The BAX variant G108V has been associated with Burkitt Lymphoma [38]. We analyzed the interaction BAX-BID in its native form and the G108V variant (mutant form) generated with Modeller [19]. At a global level, only two of SPs are slightly higher for the mutant (i.e. PAIR, ES3DC and their respective Z-scores) while the rest remain unaffected (see Additional file 14: Table S2). However, the analysis of the interface identifies the detrimental effect of the mutation, as observed in the region around residues 108–110 of BAX (see Additional file 5: Figure S4).

### Evaluation of the SPServer global and residue scores on the CASP12 benchmark

**Fig. 3:**
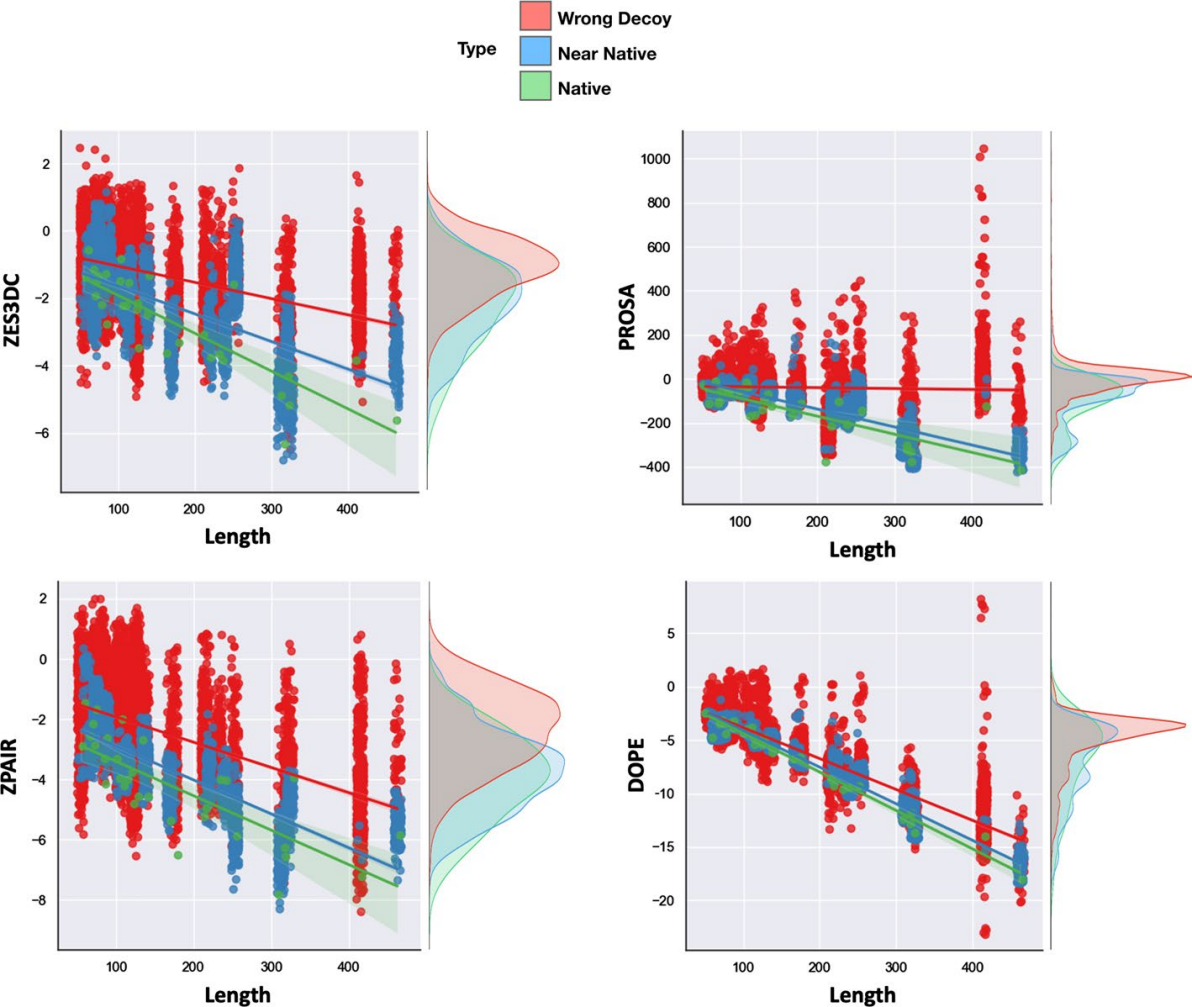
Distribution of scores for proteins in CASP12 dataset. Scores of native (green), near native (blue) and wrong decoy structures (red) are shown with respect to the protein number of residues. The figure shows in four panels the distributions of scores obtained with PROSA (Z-score of Pair potential), DOPE and the Z-scores of PAIR (ZPAIR) and ES3DC (ZES3DC). Distribution of scores independent of protein length are shown in the left of each panel

We test the SPs of the SPServer on the CASP12 [36] benchmark curated by López-Blanco et al*.* [39] (Additional file 17: Table S5). We classify the decoys of the benchmark as near-native (GDT_TS $\ge$ 65%, as defined in [40]) and wrong (GDT_TS < 65%). The final CASP12 benchmark contains 9,977 structures, of which 2,100 were classified as near-native and 7,845 as wrongly modelled, and 32 were the native structure. We compare SPServer global and local scores with those from two standard scoring programs: PROSA [6] and DOPE [41]. In Fig. 3, we show the distributions of different scores for wrongly modelled decoys, near-native decoys and native structures in the CASP12 benchmark for proteins with different length. The scoring functions distinguish between native and non-native structures, assigning lower scores to native, higher scores to near-native and much higher to wrong decoy conformations. For proteins longer than 200 residues, all scoring approaches clearly separate native, near-native and wrong conformations. However, the scores of PROSA (Z-score of Pair potential), ZES3DC (Z-score normalized ES3DC) and ZPAIR (Z-score of PAIR) are optimal to distinguish between native and non-native structures.

In the Additional file 1: Data, we include the pairwise correlations between the global (full protein) and local (per residue) scores of the SPServer scoring functions PAIR and ES3DC, and the scores of PROSA (Pair potential) and DOPE. The Pearson correlation coefficients between the potentials ZPAIR and ZES3DC and the state-of-the-art potentials PROSA and DOPE are higher than 0.6 (ranging between 0.6 and 0.72, see Additional file 15: Table S3 and Additional file 6: Figure S5). We also compared the local scores (profiles per residue) of the different scoring functions. The SPServer profiles with score PAIR are correlated with the profiles using DOPE (0.57) and PROSA (0.38) (see Additional file 16: Table S4 and Additional file 12: Figure S11).

Additionally, we compare the global Z-scores of SPServer with three quality metrics used as reference in CASP: Template Modelling (TM) score [42], Global Distance Test (GDT_TS) [43] and Quality Control Score (QCS) [44]. TM score and GDT_TS measure the quality of a model based on its similarity with the native structure. In contrast, QCS measures the quality of the model based on structural features such as the position of its secondary structure elements. Additional file 15: Table S3 and Additional file 6: Figure S5 compare ZPAIR and ZES3DC global scores with TM, GDT_TS and QCS (Pearson correlations range between − 0.44 and − 0.58). Our scores compete with other scores, such as the Z-score of PROSA or the global score of DOPE, showing similar Pearson correlations with both (ranging between − 0.1 and − 0.47), proving their utility to detect the right fold among several decoys. The comparison of scores for all the CASP12 structures can be easily visualized as scatter plots in Additional files 7–11: Figures S6–S10.

### Comparison of the SPServer interface with other protein scoring web servers

We compared the SPServer in terms of input flexibility, user-friendliness, speed and intuitive visualization of results with other state-of-the-art functional web servers for protein fold assessment (ANOLEA [14], MODFOLD6 [18], ProQ3D [17], ProSA-web [6], QMEAN [16], Verify 3D [15], VoroMQA [8]). SPServer, ANOLEA [14], PROSA-web [6] and QMEAN [16] use statistical potentials. ModFOLD6 [18] and ProQ3D [17] combine several structural features and outputs from 3rd party software into neural networks. QMEAN [16] and VERIFY 3D [15] analyze local structural features such as the secondary structure, the degree of exposure and the degree of polarity for each amino acid. VoroMQA [8] analyzes contact regions based on the study of van der Waals radius through Voronoi tessellations. The comparison is summarized in Table 1.

**Table 1:**
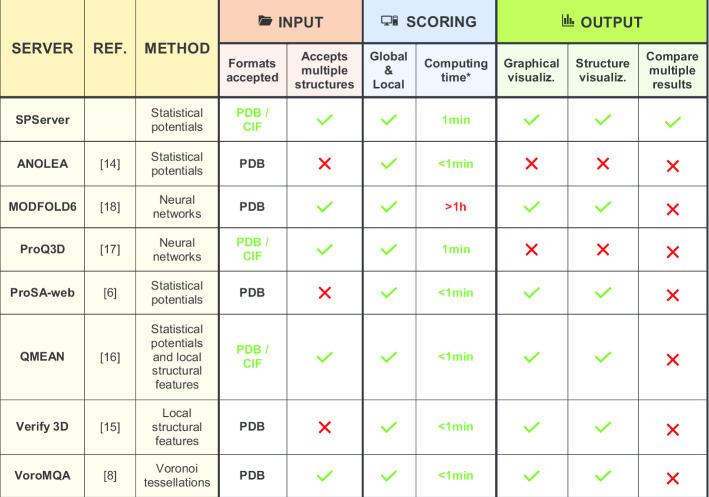
Comparison of the input, scoring and output functionalities of the SPServer and other current servers for the assessment of protein folds

*The computing time is approximated using the same structure (1mbn.pdb) and includes the queue waiting time at the moment of submission. We note that the requirement of time may have a strong dependence on the number of users and must not be used to compare the performance

In terms of input flexibility, the SPServer accepts both PDB and mmCIF formats, inputs with single or multiple structures, and does not require the sequence or the identifiers of the protein chains because it handles everything automatically. In contrast, only ProQ3D and QMEAN accept mmCIF format, and only MODFOLD6, ProQ3D, QMEAN and VoroMQA accept multiple structures.

In terms of scoring calculation, all the web servers offer both global and local scores in short time. The only web server requiring some extra time of calculation is MODFOLD6, as it integrates different scoring functions and the use of neural networks.

Finally, in terms of intuitive visualization of the results, most web servers offer clear plots for the analysis of local scores. They also provide a tool to visualize the structure, where the residues are colored according to their local score. Still, only the SPServer provides interactive tools to easily compare the local scores of multiple structures; the local scores can be visualized together in the same plot and smoothed or shifted according to the user’s preferences. Additionally, none of the methods reviewed provide tools to score the quality of the interface of PPIs.

## Conclusions

The SPServer facilitates the quality assessment of both protein folds and protein–protein interaction structures in an easy-to-use web server. The quality assessment of the structures is obtained with Split-Statistical Potentials scoring functions that handle several terms associated with the structural local features of the amino acid environments. They are obtained from the analysis of empirical structures: different terms are taken into account such as pairs of interacting residues, solvent accessibility or type of secondary structure. The Split-Statistical Potentials have been tested on the CASP12 dataset and distinguish successfully native structures from wrong decoys. Moreover, the resulting scores are highly correlated with those from reference scoring functions such as PROSA and DOPE. While the other state-of-the-art web servers only show the local scores of the structures in a plot, the SPServer permits to compare different local score profiles simultaneously. This is done in an interactive plot where the scores can be smoothed or shifted to facilitate the analysis and visualization. Thanks to these analytical tools, we can use the SPServer to compare the quality of different protein models and protein–protein interactions, or to understand better the structural effect of a mutation both on the fold and the binding.

## Availability and requirements

- Project name: SPServer.
- Project home page: https://sbi.upf.edu/spserver.
- Operating system(s): Platform independent.
- Programming language: PHP, JavaScript, Python.
- Other requirements: Chrome, Safari, Firefox or any other modern browser.
- License: Open Source.
- Any restrictions to use by non-academics: None.

## Supplementary information

**Additional file 1**. Data.

**Additional file 2**. ** Figure S1**: Identification of steric crashes using GEPOL approach to calculate the surface. The two atoms are represented as light blue and light brown circles. The normal and position vectors are shown both in a case where there is no steric crash (a), and there is a steric crash (b). In the case (a) both vectors form and acute angle (i.e. < 90°) while in the case (b) they form an obtuse angle (i.e. > 90), and thus the sign of the two dot products will be negative.

**Additional file 3**. ** Figure S2**: Residue scores of the native structure of Cysteine synthase A (green), the near-native model (blue) and the wrong model (red). The curves represent the smoothed PAIR scores with a sliding window of value 10.

**Additional file 4**. ** Figure S3**: Difference between the residue scores of the native structure (reference) and the near-native (blue) and wrong (red) models. The curves represent the smoothed PAIR scores with a sliding window of value 10.

**Additional file 5**. **Figure S4**: Local scores map of the interface of the interaction between BAX (Receptor) and BID (Ligand). Large cells are used for local scores (statistic energy) of the wildtype structure and upper (smaller) squares are for the mutant. Energies are shown by colors, from high (red) to low (blue), indicating the range in the label at the bottom. The scores are calculated with the PAIR potential, using a sliding window of 1 to smooth, being the optimal interactions those with most negative energy.

**Additional file 6**. ** Figure S5**: Mean Pearson correlation values of the comparison between the global scores of the SPServer (ZES3DC and ZPAIR), DOPE and PROSA (Pair Z-score) potentials, and TM, GDT_TS and QCS quality metrics for the structures of CASP12 benchmark. The correlation values are extracted after performing a bootstrapping strategy of 1000 repetitions (described above). The Pearson correlation values of TM score, GDT_TS and QCS are negative because their score is higher when the model is more similar to the native structure (the opposite of the statistical potentials).

**Additional file 7. Figure S6**: Scatter plots of the global scores of the SPServer potentials ZES3DC (a) and ZPAIR (b) with respect to PROSA (Z-score of Pair potential) for the structures of the CASP12 benchmark.

**Additional file 8**.** Figure S7**: Scatter plots of the global scores of the SPServer potentials ZES3DC (a) and ZPAIR (b) with respect to DOPE for the structures of the CASP12 benchmark.

**Additional file 9**. ** Figure S8**: Scatter plots of the global scores of the SPServer potentials ZES3DC (a) and ZPAIR (b) with respect to GDT_TS for the structures of the CASP12 benchmark.

**Additional file 10**. **Figure S9**: Scatter plots of the global scores of the SPServer potentials ZES3DC (a) and ZPAIR (b) with respect to TM score for the structures of the CASP12 benchmark.

**Additional file 11**.** Figure S10**: Scatter plots of the global scores of the SPServer potentials ZES3DC (a) and ZPAIR (b) with respect to QCS for the structures of the CASP12 benchmark.

**Additional file 12**. ** Figure S11**: Histograms showing the residue correlations between the SPServer scoring functions (ES3DC and PAIR) and the PROSA (Pair) and DOPE scoring functions. Each correlation value corresponds to the correlation of all the residue scores of a structure from the CASP12 benchmark.

**Additional file 13**. ** Table S1**: Global scores of the native structure of Cysteine synthase A and two predicted structural models.

**Additional file 14**. **Table S2**: Global scores of the native structure of Cysteine synthase A and two of its models.

**Additional file 15**. ** Table S3**: Comparison between global and quality metrics for the structures of CASP12 benchmark.

**Additional file 16**. ** Table S4**: Comparison of local (residue) profiles between SPServer and state-of-art methods DOPE and PROSA for the structures of CASP12 benchmark.

**Additional file 17**. **Table S5**: SPServer global Z-scores (ZES3DC, ZPAIR), PROSA (Pair Z-score), DOPE score, GDT_TS, TM score and QCS of the structures of the CASP12 benchmark.

## Data Availability

The web server can be found at https://sbi.upf.edu/spserver. The standalone software can be found at https://github.com/structuralbioinformatics/SPServer.

## Abbreviations

PPIs: Protein–protein interactions
SPs: Split-statistical potentials
SPServer: Split-statistical potentials server
