# Supplementary material for: SPServer: split-statistical potentials for the analysis of protein structures and protein–protein interactions
Source: BMC Bioinformatics. 2021 Jan 6;22:4. doi: 10.1186/s12859-020-03770-5 (PMC7788957; doi:10.1186/s12859-020-03770-5)
Supplement: Supplementary file 1 — Additional file 1. Data. [file 12859_2020_3770_MOESM1_ESM.docx]

**Supplementary material for:**

**SPServer: Split-statistical potentials for the analysis of protein structures and protein-protein interactions**

Joaquim Aguirre-Plans, Alberto Meseguer, Ruben Molina-Fernández, Manuel Alejandro Marín-López, Gaurav Jumde, Kevin Casanova, Jaume Bonet, Oriol Fornes, Narcis Fernandez-Fuentes and Baldo Oliva

[**Identification of steric clashes in protein interfaces** 2](#_Toc51235880)

[**Study of the structural models of Cysteine synthase A** 2](#_Toc51235881)

[**Study of a mutation in the interaction between BAX and BID** 4](#_Toc51235882)

[**Comparison of the SPServer global and local (profile) scores with reference scoring functions using a CASP12 benchmark** 5](#_Toc51235883)

[**Legends to Supplementary Data** 7](#_Toc51235884)

[**References** 9](#_Toc51235885)

# **Identification of steric clashes in protein interfaces**

As part of the SPServer analyses of protein complexes, we have implemented a method to identify atomic clashes in protein interfaces based on the program GEPOL [1]. GEPOL approximates the molecular surface of proteins by triangular tessellation of the atoms, i.e. the surface of atoms is represented by a set of triangles. On a first step, we identify the interface of the complex by filtering those surface regions that do not change when comparing bound and unbound conformations. Subsequently, the triangles enclosed in the interface(s) of the complex are extracted, and these are used to identify steric crashes.

For each pair of triangles from different protein chains and a distance smaller than 0.3 Angstroms we compute: (i) the *normal vector*; (ii) a *position vector* calculated as the difference between the centers of the two triangles; and (iii) the *dot product* between the normal vector and the position unit vector. By comparing the sign of the dot product, it is possible to identify whether the two given atoms are indeed overlapping, i.e. steric crash (see **Supplementary Figure S1**). This information is provided in the summary table under the column ‘crashes’ with a yes/no value. If the value is yes, then a color gradient is shown together with a clickable pop-up window identifying the residues and atoms as well as the penetration area accounting for the steric hindrances.

# **Study of the structural models of Cysteine synthase A**

CASP12 is a community-wide experiment to evaluate model estimation accuracy methods [2]. We selected from CASP12 one of the 82 prediction targets released: the structure of Cysteine synthase A from *E. coli* (target code **T0861**, PDB code 5j5v) [3]. For this target, 181 models were released (the models can be downloaded at <http://predictioncenter.org/download_area/CASP12/predictions/>). We selected 2 of these models: the top-ranked model also called “near-native model” (T0861TS275_2) and the bottom-ranked model also called wrong model or “decoy” (T0861TS321_1).

The ranking of models can be downloaded at <http://predictioncenter.org/download_area/CASP12/SUMMARY_TABLES/>.

We compared the native structure of both models using the SPServer. The global scores show that the near-native model has lower scores than the wrong model (in PAIR, ECOMB, E3DC, E3D, ZPAIR, ZES3DC and ZE3DC; see **Supplementary Table S1**).

In the residue scores plot we can see how the curve of the near-native model is almost identical to the curve of the native model. Contrarily, the curve of the wrong model has higher values (see Supplementary Figure S2). The difference between the residues’ energy between the native structure and the two models can be highlighted by plotting the difference between their scores. We see that the score difference from the native and the near-native structures is always close to 0. Conversely, the difference between the near-native and the wrong decoys scores is higher (see Supplementary Figure S3). Thus, the near-native model is a more reliable and similar to the native structure than the wrong one.

The results can be explored in more detail using the job ID **fold_cysteine_synthase** or in following link: <http://aleph.upf.edu/spserver/index.php/results/job/fold_cysteine_synthase.html>

# **Study of a mutation in the interaction between BAX and BID**

We modeled BAX – BID interaction using as template the PDB structure 4bd2 [4], containing the interaction of BAX and BID. We modeled two variants of full length BAX sequence: a wildtype model and a model with the G108V mutation and reconstructed the interaction with BID using the structure of the protein complexes above using MODELLER [5].

We compared the scores of the two models using the SPServer. Some of the global scores obtained are higher in the case of the mutant G108V (PAIR, ES3DC and their Z-scores, see **Supplementary Table S2**). By checking the local score maps we can observe an increase in the scores, i.e. higher energy, in the region of residues 108 to 110 (BAX) and 18 to 30 (BID) indicating that the mutation has a detrimental effect in this region of the interaction (see Supplementary Figure S4).

The results can be explored in more detail using the job ID **ppi_bax_bid** or in the following link: <http://aleph.upf.edu/spserver/index.php/results/job/ppi_bax_bid.html>

# **Comparison of the SPServer global and local (profile) scores with reference scoring functions using a CASP12 benchmark**

We compare two scoring functions of the SPServer (PAIR and ES3DC) with two reference scoring functions (PROSA [6] and DOPE [7]) and three reference quality metrics (TM score [8], GDT_TS [9] and QCS [10]). We calculate the correlation between the global and local scores of the different scoring functions for native, near-native and decoy structures of the CASP12 dataset.

***CASP12 benchmark***

We use as reference dataset the CASP12 benchmark curated by *López-Blanco et al.* [11]. This dataset contains all the CASP12 decoys trimmed to single domains. The targets without any decoy of GDT_TS higher than 50% are discarded. The benchmark can be downloaded using this url: <https://chaconlab.org/modeling/korp/down-korp/item/rcd-v111-linux-copy>

We classify the decoys with GDT_TS $\geq$ 65% as near-native and the decoys with GDT_TS < 65% as wrong. The final CASP12 benchmark contains 9,977 structures, of which 2,100 were classified as near-native and 7,845 as wrongly modelled, and 32 were the native structure. The CASP12 benchmark, with the scores for all the scoring functions (ZES3DC, ZPAIR, PROSA, DOPE, GDT_TS, TM score, QCS) is described in **Supplementary Table S5**.

***Bootstrapping strategy***

We applied a bootstrapping strategy to calculate the correlations between SPServer Z-scores and the rest of the scoring functions. By applying bootstrapping, we avoid the unbalance in the number of native, near-native and wrong structures. The strategy is described as follows:

1. For each target in the CASP12 benchmark, we use its native structure and randomly select one near-native and one wrong decoy.
2. We calculate the Pearson correlation between several scores used to evaluate all the selected structures.
3. We repeat the previous two steps 1,000 times.
4. We calculate the mean and standard deviation of the correlations.

***Global score correlations***

We calculate the correlation between the global scores of several scoring functions by bootstrapping. Results are summarized in **Supplementary Table S3** and **Supplementary Figure S5**. Additionally, we plot the SPServer Z-scores to compare the values of the scoring functions applied on the total set of 9977 structures (native, near-native and wrong decoys) of the CASP12 benchmark in **Supplementary Figure S6** **to** **S10**.

***Local score correlations***

We calculate the correlation between the local scores of the different scoring functions, classifying the structures in native, near-native and wrong. Briefly, for each structure we calculate the correlation of the residue scores between the different scoring functions. **Supplementary Table S4** summarizes the mean correlation of the local scores (per residue). **Supplementary Figure S11** shows the histogram distribution of Pearson correlations between the local scores of PAIR, ES3DC and the scores of the potentials of DOPE and PROSA Pair.

# **Legends to Supplementary Data**

**Supplementary Table S1. Global scores of the native structure of Cysteine synthase A and two predicted structural models**.

**Supplementary Table S2. Global scores of the native structure of Cysteine synthase A and two of its models.**

**Supplementary Table S3: Comparison between global and quality metrics for the structures of CASP12 benchmark.**

**Legend Table S3: Mean Pearson correlation values and standard deviations of the comparison between the global scores of the SPServer (ZES3DC and ZPAIR), DOPE and PROSA (Pair Z-score) potentials, and TM, GDT_TS and QCS quality metrics for the structures of CASP12 dataset**. The correlation values are obtained by bootstrapping strategy using 1000 repetitions (see above). The Pearson correlation values between the scoring functions (using SPServer, DOPE and PROSA) and TM score, GDT_TS and QCS are negative because these metrics increase (from 0 to 1) with the quality of the structure, while scores are energy functions that decrease lower than 0 (being the lowest energy the best conformation).

**Supplementary Table S4: Comparison of local (residue) profiles between SPServer and state-of-art methods DOPE and PROSA for the structures of CASP12 benchmark.**

**Legend Table S4: Mean Pearson correlation values between the local (residue) scores of SPServer and state-of-art methods DOPE and PROSA for all structures of the CASP12 benchmark**. Each correlation value corresponds to the correlation of all the residue scores of a structure from the CASP12 benchmark.

**Supplementary Figure S1. Identification of steric crashes using GEPOL approach to calculate the surface**. The two atoms are represented as light blue and light brown circles. The normal and position vectors are shown both in a case where there is no steric crash (a), and there is a steric crash (b). In the case (a) both vectors form and acute angle (i.e. < 90º) while in the case (b) they form an obtuse angle (i.e. >90), and thus the sign of the two dot products will be negative.

**Supplementary Figure S2. Residue scores of the native structure of Cysteine synthase A (green), the near-native model (blue) and the wrong model (red).** The curves represent the smoothed PAIR scores with a sliding window of value 10.

**Supplementary Figure S3. Difference between the residue scores of the native structure (reference) and the near-native (blue) and wrong (red) models.** The curves represent the smoothed PAIR scores with a sliding window of value 10.

**Supplementary Figure S4. Local scores map of the interface of the interaction between BAX (Receptor) and BID (Ligand).** Large cells are used for local scores (statistic energy) of the wildtype structure and upper (smaller) squares are for the mutant. Energies are shown by colors, from high (red) to low (blue), indicating the range in the label at the bottom. The scores are calculated with the PAIR potential, using a sliding window of 1 to smooth, being the optimal interactions those with most negative energy.

**Supplementary Figure S5.** **Mean Pearson correlation values of the comparison between the global scores of the SPServer (ZES3DC and ZPAIR), DOPE and PROSA (Pair Z-score) potentials, and TM, GDT_TS and QCS quality metrics for the structures of CASP12 benchmark**. The correlation values are extracted after performing a bootstrapping strategy of 1000 repetitions (described above). The Pearson correlation values of TM score, GDT_TS and QCS are negative because their score is higher when the model is more similar to the native structure (the opposite of the statistical potentials).

**Supplementary Figure S6.** **Scatter plots of the global scores of the SPServer potentials ZES3DC (a) and ZPAIR (b) with respect to PROSA (Z-score of Pair potential) for the structures of the CASP12 benchmark**.

**Supplementary Figure S7.** **Scatter plots of the global scores of the SPServer potentials ZES3DC (a) and ZPAIR (b) with respect to DOPE for the structures of the CASP12 benchmark**.

**Supplementary Figure S8.** **Scatter plots of the global scores of the SPServer potentials ZES3DC (a) and ZPAIR (b) with respect to GDT_TS for the structures of the CASP12 benchmark**.

**Supplementary Figure S9.** **Scatter plots of the global scores of the SPServer potentials ZES3DC (a) and ZPAIR (b) with respect to TM score for the structures of the CASP12 benchmark**.

**Supplementary Figure S10.** **Scatter plots of the global scores of the SPServer potentials ZES3DC (a) and ZPAIR (b) with respect to QCS for the structures of the CASP12 benchmark**.

**Supplementary Figure S11. Histograms showing the residue correlations between the SPServer scoring functions (ES3DC and PAIR) and the PROSA (Pair) and DOPE scoring functions**. Each correlation value corresponds to the correlation of all the residue scores of a structure from the CASP12 benchmark.

# **References**

1. Pascual‐ahuir JL, Silla E, Tuñon I. GEPOL: An improved description of molecular surfaces. III. A new algorithm for the computation of a solvent‐excluding surface. J Comput Chem. 1994.

2. Kryshtafovych A, Monastyrskyy B, Fidelis K, Schwede T, Tramontano A. Assessment of model accuracy estimations in CASP12. Proteins Struct Funct Bioinforma. 2017;86 July:1–16. doi:10.1002/prot.25371.

3. Johnson PM, Beck CM, Morse RP, Garza-Sánchez F, Low DA, Hayes CS, et al. Unraveling the essential role of CysK in CDI toxin activation. Proc Natl Acad Sci. 2016.

4. Czabotar PE, Westphal D, Dewson G, Ma S, Hockings C, Fairlie WD, et al. Bax crystal structures reveal how BH3 domains activate Bax and nucleate its oligomerization to induce apoptosis. Cell. 2013;152:519–31. doi:10.1016/j.cell.2012.12.031.

5. Webb B, Sali A. Comparative protein structure modeling using MODELLER. Curr Protoc Bioinforma. 2016.

6. Wiederstein M, Sippl MJ. ProSA-web: Interactive web service for the recognition of errors in three-dimensional structures of proteins. Nucleic Acids Res. 2007;35 SUPPL.2:407–10.

7. Shen M-Y, Sali A. Statistical potential for assessment and prediction of protein structures. Protein Sci. 2006;15:2507–24.

8. Zhang Y, Skolnick J. Scoring function for automated assessment of protein structure template quality. Proteins Struct Funct Genet. 2004;57:702–10. doi:10.1002/prot.20264.

9. Zemla A. LGA: A method for finding 3D similarities in protein structures. Nucleic Acids Res. 2003;31:3370–4.

10. Cong Q, Kinch LN, Pei J, Shi S, Grishin VN, Li W, et al. An automatic method for CASP9 free modeling structure prediction assessment. Bioinformatics. 2011;27:3371–8.

11. López-Blanco JR, Chacón P. KORP: Knowledge-based 6D potential for fast protein and loop modeling. Bioinformatics. 2019;35:3013–9.
