# Supplementary figures and images for: SPServer: split-statistical potentials for the analysis of protein structures and protein–protein interactions
Source: BMC Bioinformatics. 2021 Jan 6;22:4. doi: 10.1186/s12859-020-03770-5 (PMC7788957; doi:10.1186/s12859-020-03770-5)

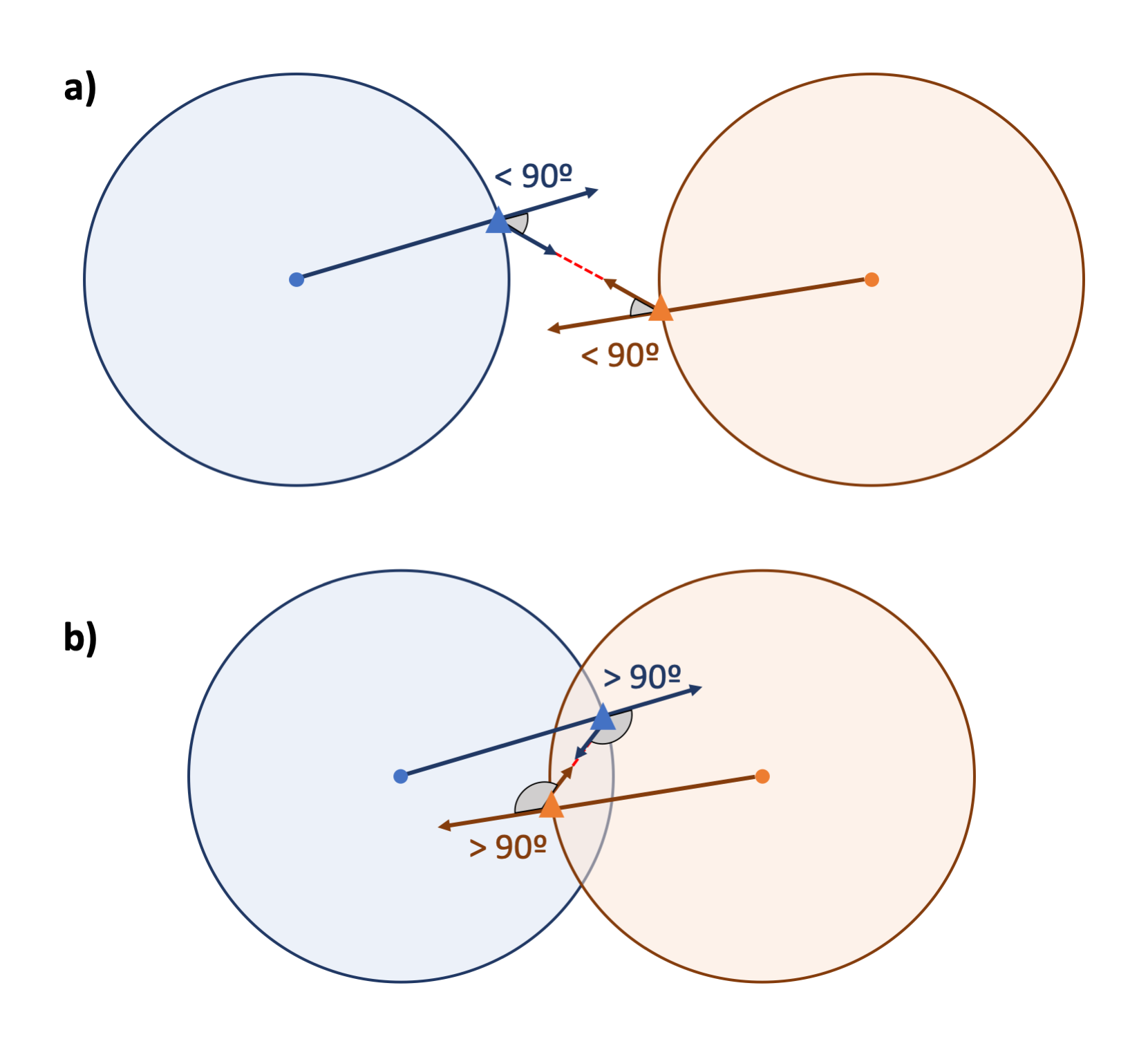

Supplement: Supplementary file 2 — Additional file 2. Figure S1: Identification of steric crashes using GEPOL approach to calculate the surface. The two atoms are represented as light blue and light brown circles. The normal and position vectors are shown both in a case where there is no steric crash (a), and there is a steric crash (b). In the case (a) both vectors form and acute angle (i.e. < 90°) while in the case (b) they form an obtuse angle (i.e. > 90), and thus the sign of the two dot products will be negative. [file 12859_2020_3770_MOESM2_ESM.png]

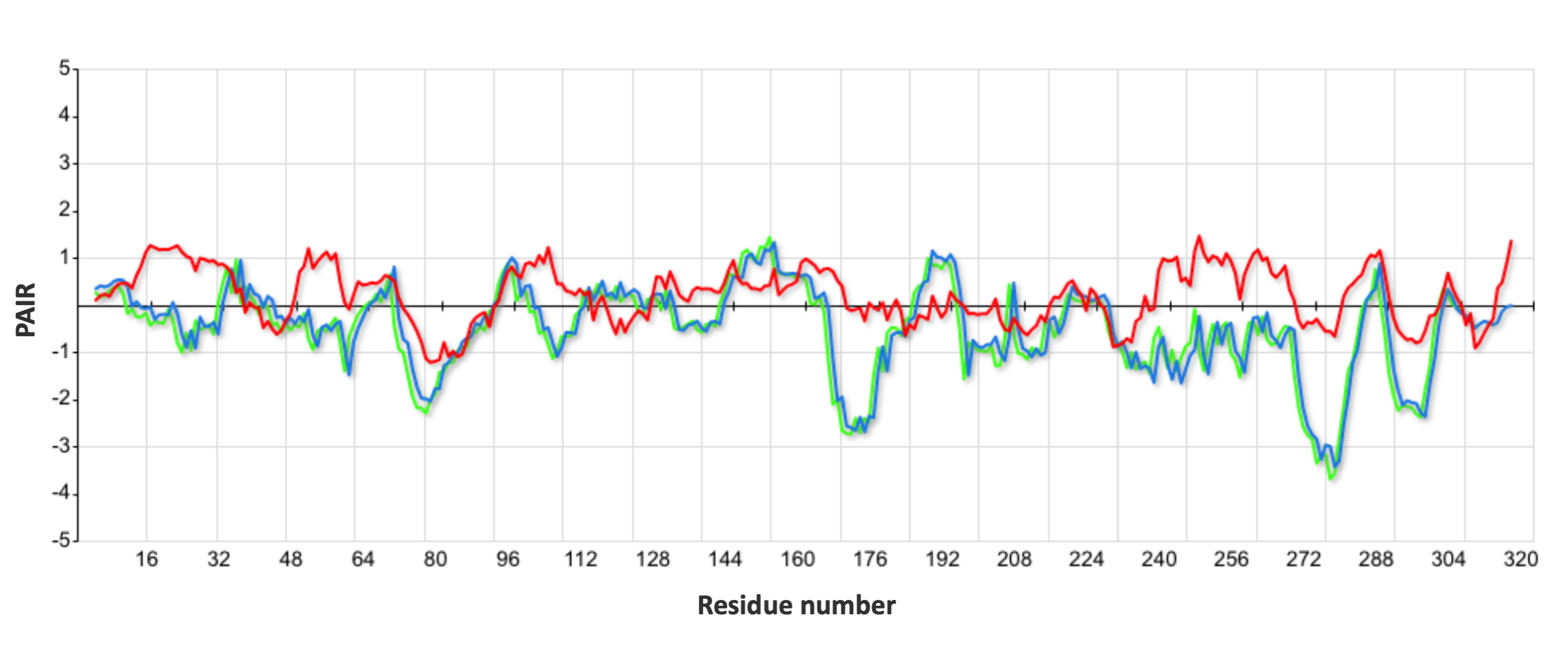

Supplement: Supplementary file 3 — Additional file 3. Figure S2: Residue scores of the native structure of Cysteine synthase A (green), the near-native model (blue) and the wrong model (red). The curves represent the smoothed PAIR scores with a sliding window of value 10. [file 12859_2020_3770_MOESM3_ESM.png]

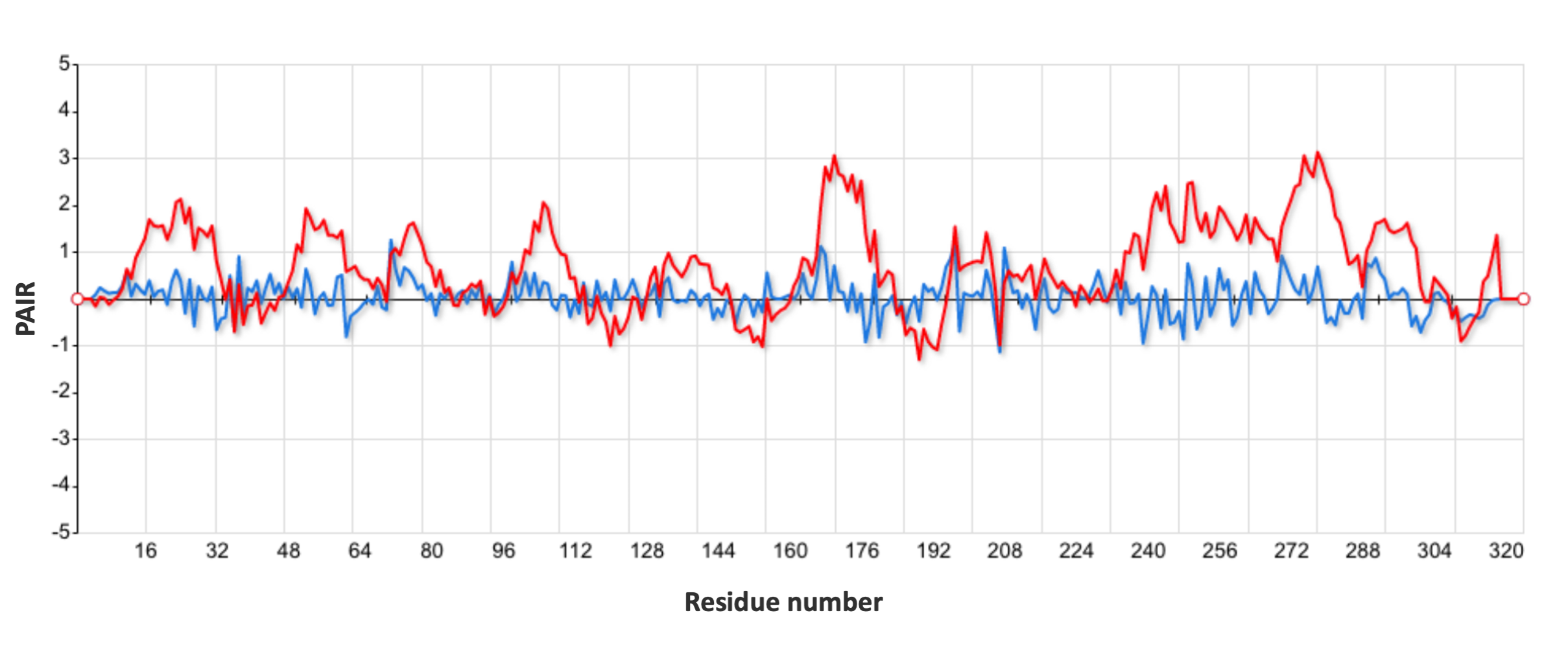

Supplement: Supplementary file 4 — Additional file 4. Figure S3: Difference between the residue scores of the native structure (reference) and the near-native (blue) and wrong (red) models. The curves represent the smoothed PAIR scores with a sliding window of value 10. [file 12859_2020_3770_MOESM4_ESM.png]

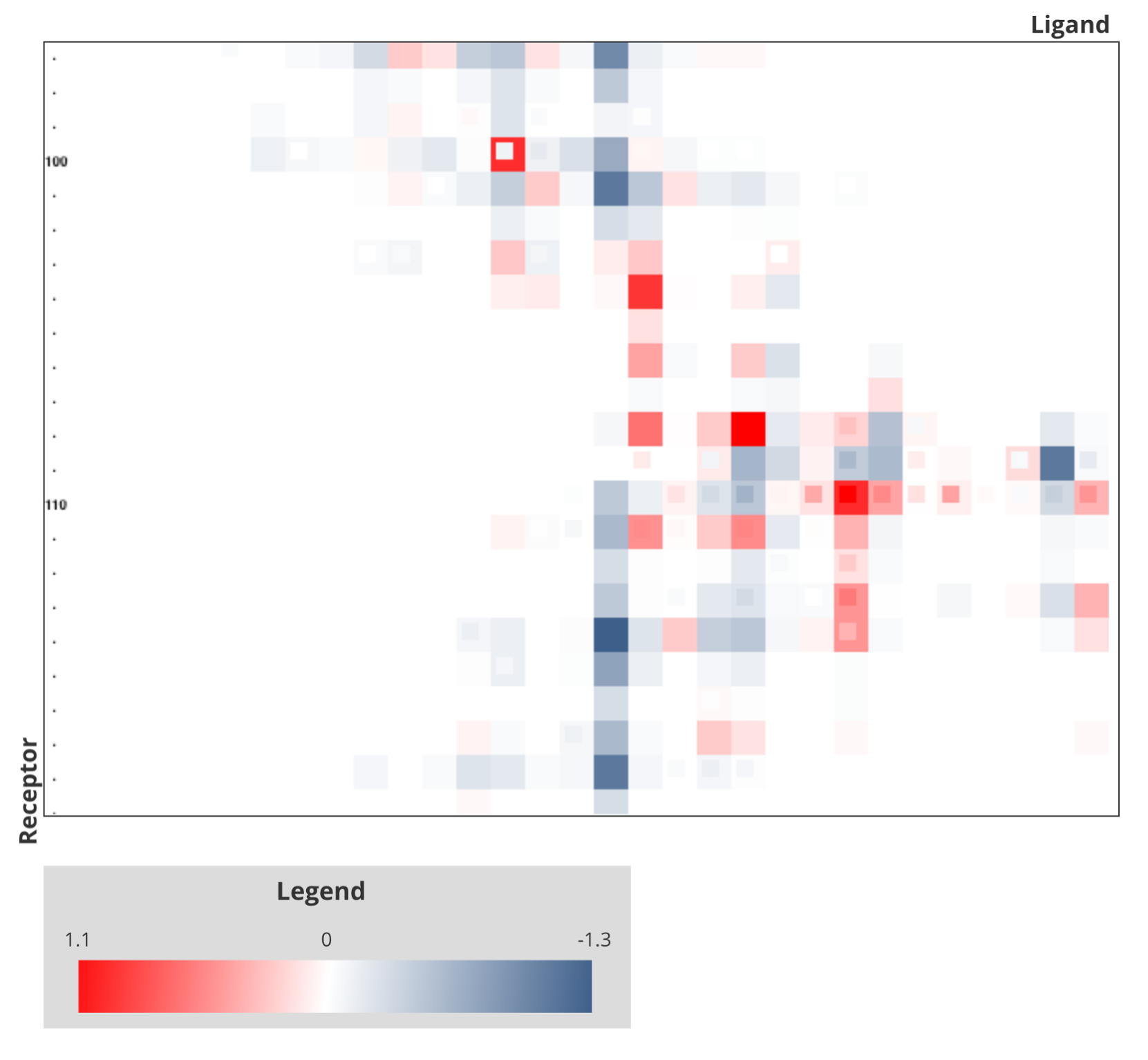

Supplement: Supplementary file 5 — Additional file 5. Figure S4: Local scores map of the interface of the interaction between BAX (Receptor) and BID (Ligand). Large cells are used for local scores (statistic energy) of the wildtype structure and upper (smaller) squares are for the mutant. Energies are shown by colors, from high (red) to low (blue), indicating the range in the label at the bottom. The scores are calculated with the PAIR potential, using a sliding window of 1 to smooth, being the optimal interactions those with most negative energy. [file 12859_2020_3770_MOESM5_ESM.png]

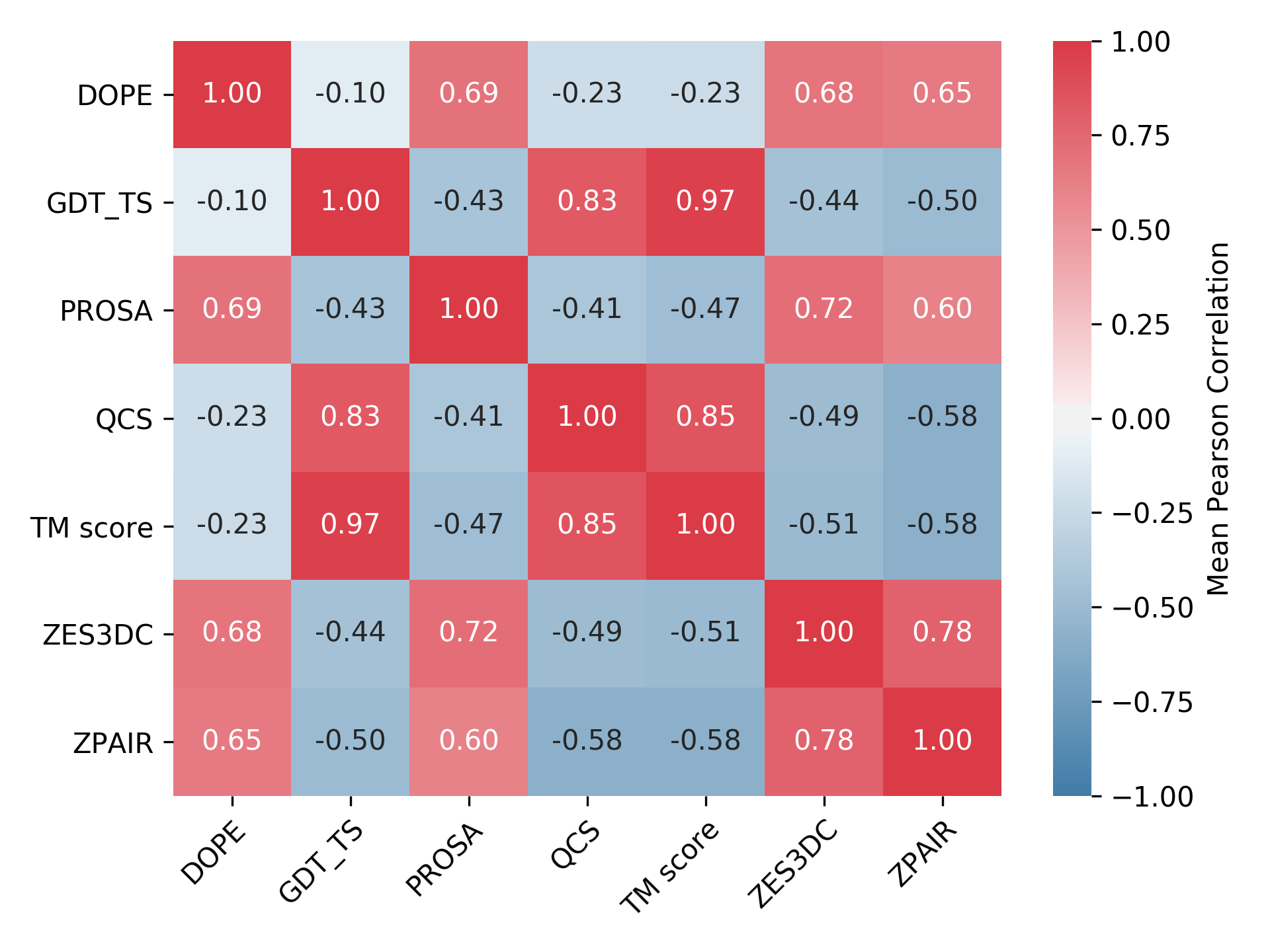

Supplement: Supplementary file 6 — Additional file 6. Figure S5: Mean Pearson correlation values of the comparison between the global scores of the SPServer (ZES3DC and ZPAIR), DOPE and PROSA (Pair Z-score) potentials, and TM, GDT_TS and QCS quality metrics for the structures of CASP12 benchmark. The correlation values are extracted after performing a bootstrapping strategy of 1000 repetitions (described above). The Pearson correlation values of TM score, GDT_TS and QCS are negative because their score is higher when the model is more similar to the native structure (the opposite of the statistical potentials). [file 12859_2020_3770_MOESM6_ESM.png]

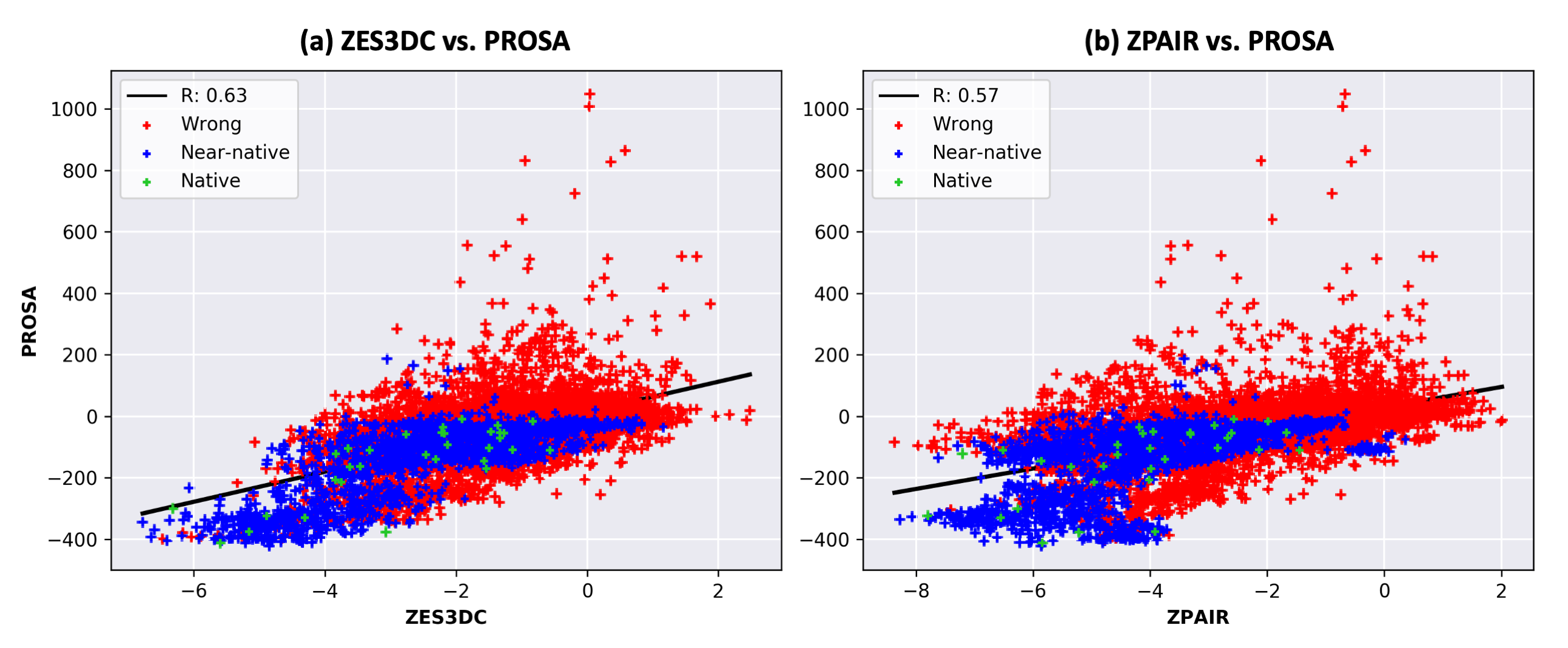

Supplement: Supplementary file 7 — Additional file 7. Figure S6: Scatter plots of the global scores of the SPServer potentials ZES3DC (a) and ZPAIR (b) with respect to PROSA (Z-score of Pair potential) for the structures of the CASP12 benchmark. [file 12859_2020_3770_MOESM7_ESM.png]

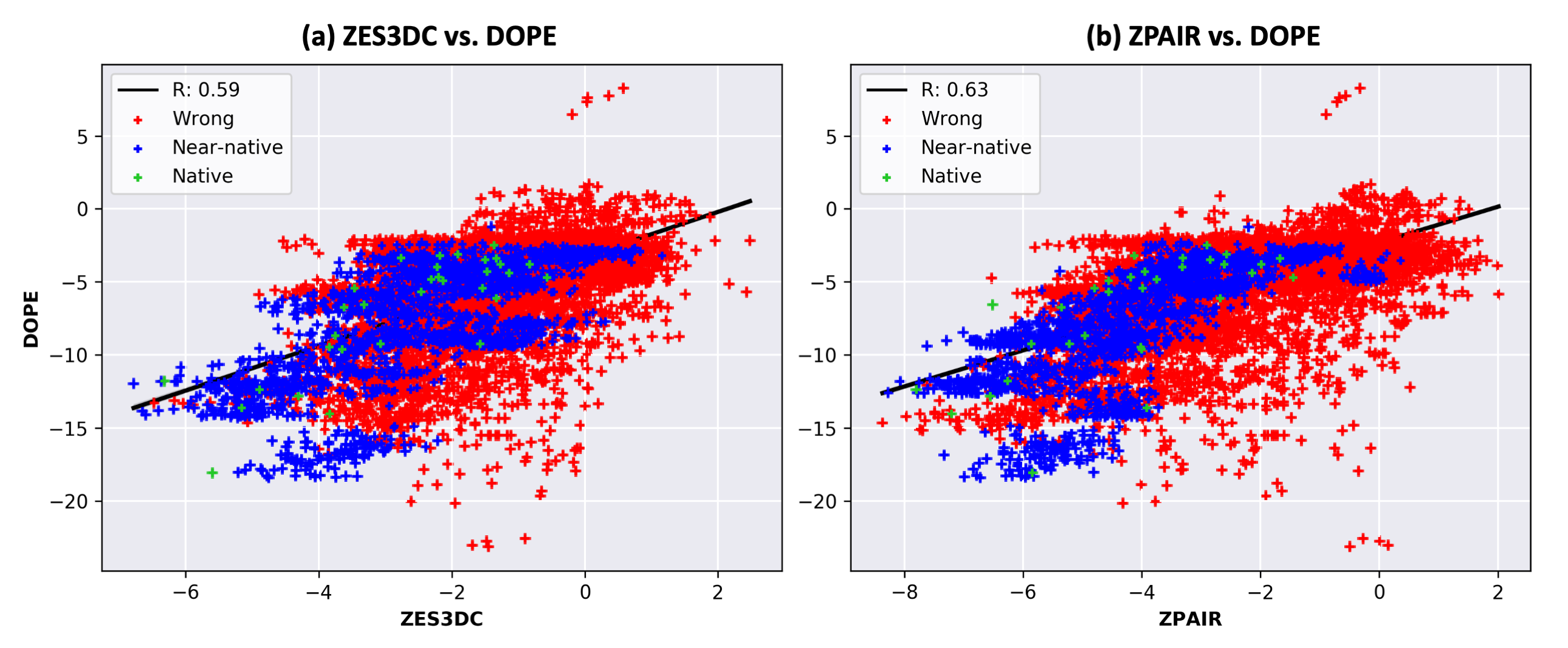

Supplement: Supplementary file 8 — Additional file 8. Figure S7: Scatter plots of the global scores of the SPServer potentials ZES3DC (a) and ZPAIR (b) with respect to DOPE for the structures of the CASP12 benchmark. [file 12859_2020_3770_MOESM8_ESM.png]

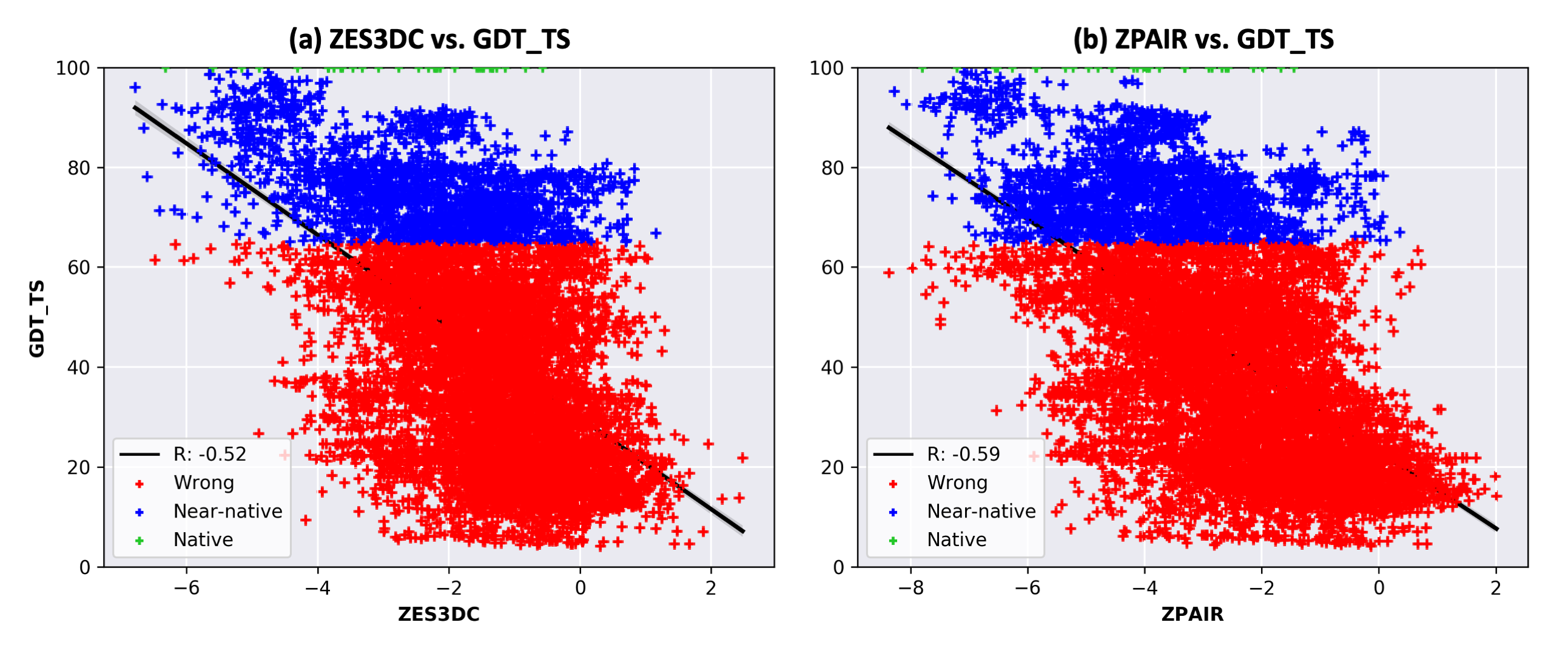

Supplement: Supplementary file 9 — Additional file 9. Figure S8: Scatter plots of the global scores of the SPServer potentials ZES3DC (a) and ZPAIR (b) with respect to GDT_TS for the structures of the CASP12 benchmark. [file 12859_2020_3770_MOESM9_ESM.png]

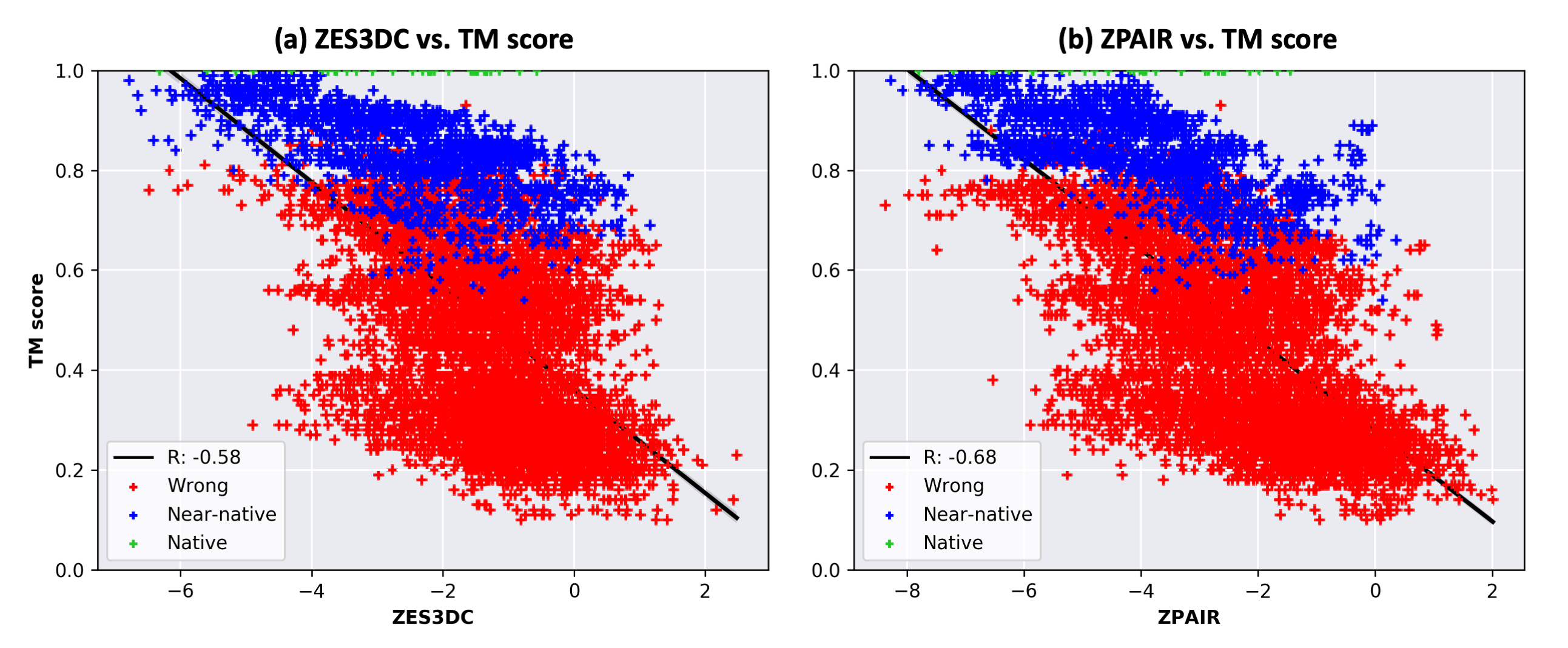

Supplement: Supplementary file 10 — Additional file 10. Figure S9: Scatter plots of the global scores of the SPServer potentials ZES3DC (a) and ZPAIR (b) with respect to TM score for the structures of the CASP12 benchmark. [file 12859_2020_3770_MOESM10_ESM.png]

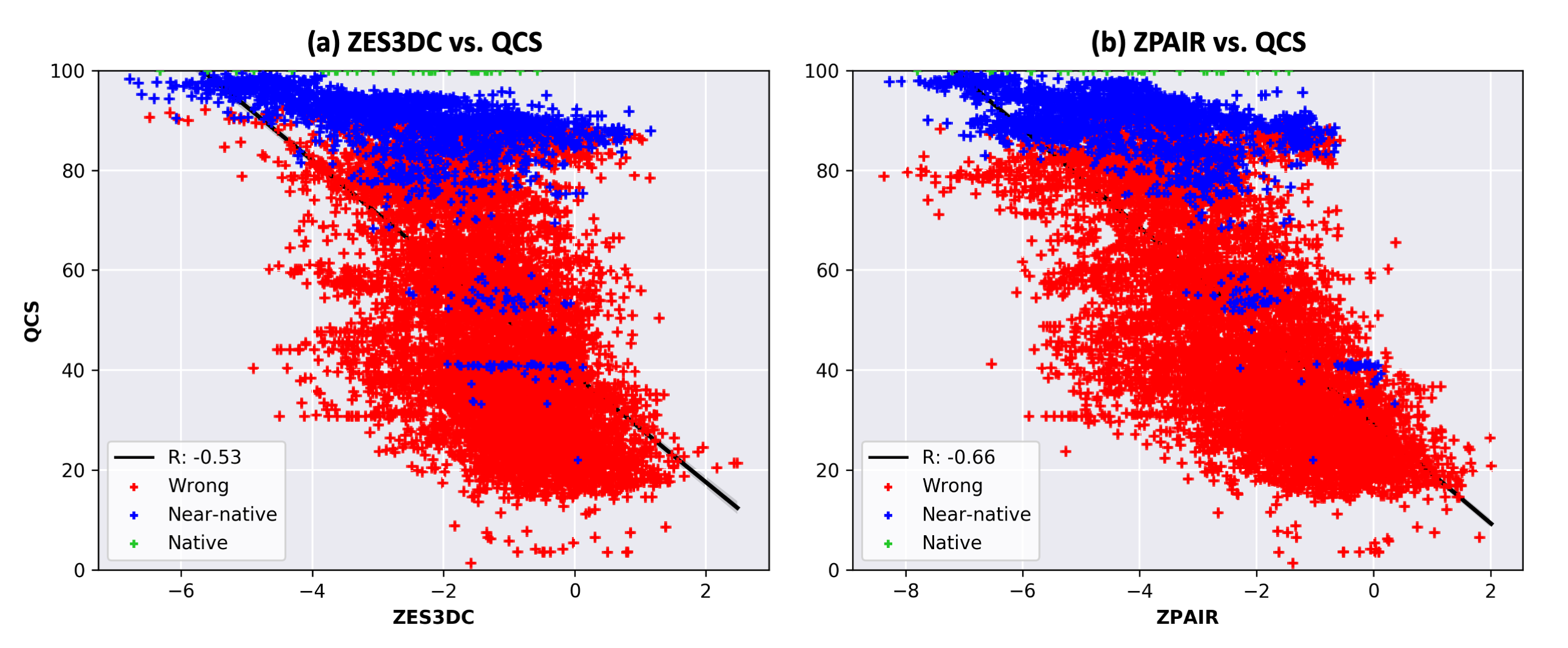

Supplement: Supplementary file 11 — Additional file 11. Figure S10: Scatter plots of the global scores of the SPServer potentials ZES3DC (a) and ZPAIR (b) with respect to QCS for the structures of the CASP12 benchmark. [file 12859_2020_3770_MOESM11_ESM.png]

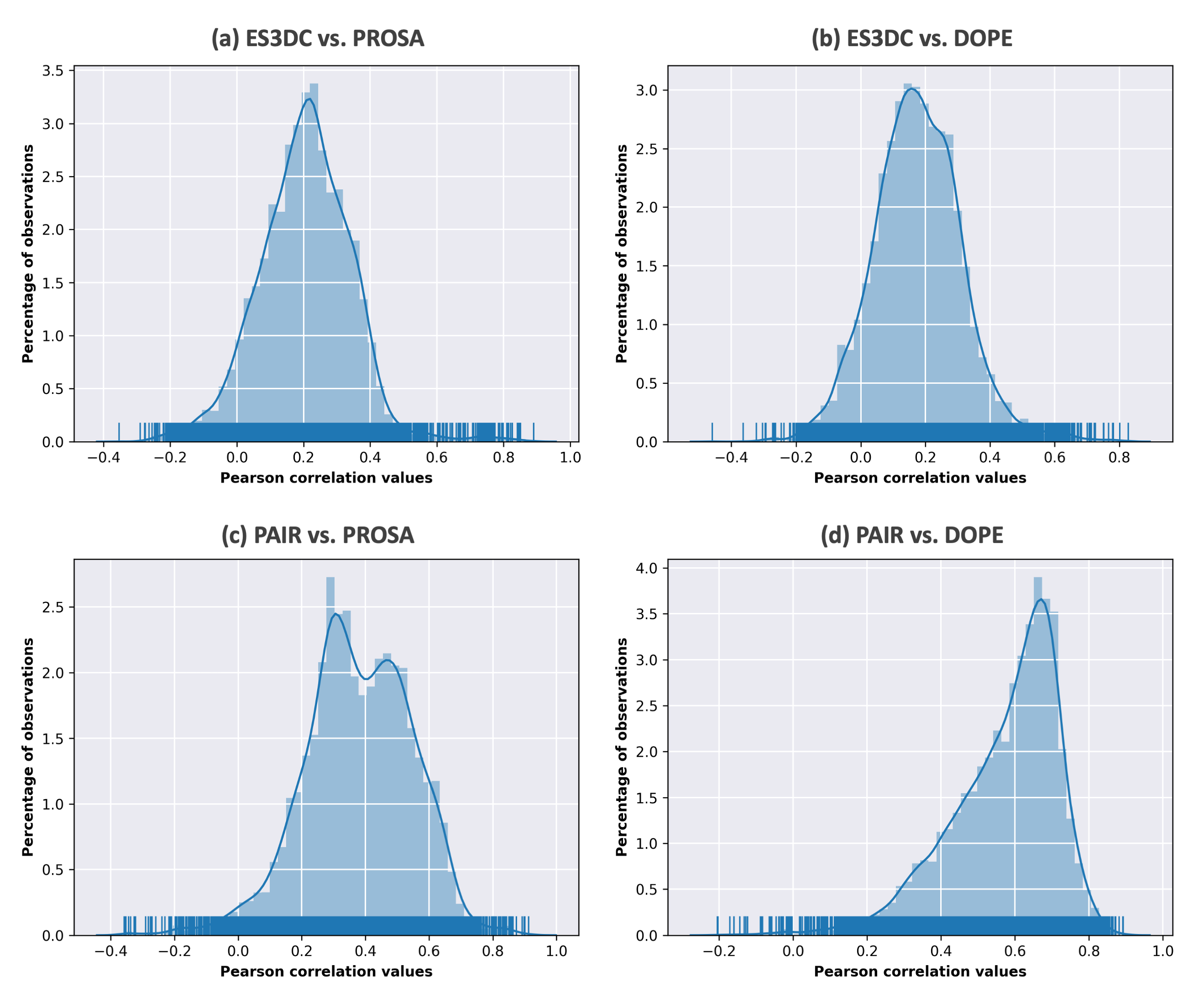

Supplement: Supplementary file 12 — Additional file 12. Figure S11: Histograms showing the residue correlations between the SPServer scoring functions (ES3DC and PAIR) and the PROSA (Pair) and DOPE scoring functions. Each correlation value corresponds to the correlation of all the residue scores of a structure from the CASP12 benchmark. [file 12859_2020_3770_MOESM12_ESM.png]
