# Supplementary material for: SPServer: split-statistical potentials for the analysis of protein structures and protein–protein interactions
Source: BMC Bioinformatics. 2021 Jan 6;22:4. doi: 10.1186/s12859-020-03770-5 (PMC7788957; doi:10.1186/s12859-020-03770-5)
Supplement: Supplementary file 13 — Additional file 13. Table S1: Global scores of the native structure of Cysteine synthase A and two predicted structural models. [file 12859_2020_3770_MOESM13_ESM.docx]

**Supplementary Table S1. Global scores of the native structure of Cysteine synthase A and two predicted structural models**.

| **Fold** | **PAIR** | **ECOMB** | **ES3DC** | **ELOCAL** | **E3DC** | **E3D** | **ZPAIR** | **ZECOMB** | **ZES3DC** | **ZELOCAL** | **ZE3DC** |
| --- | --- | --- | --- | --- | --- | --- | --- | --- | --- | --- | --- |
| **Native** | -80.69 | -6229 | -45.33 | 30467 | 29.91 | -36681 | -6.80 | -3.09 | -5.26 | -2.64 | -6.24 |
| **Near-native** | -71.63 | -6448 | -43.42 | 30301 | 20.94 | -36728 | -5.98 | -3.59 | -4.28 | -3.17 | -6.43 |
| **Wrong** | 36.76 | -3516 | 57.19 | 24823 | 74.14 | -28471 | -2.76 | -3.66 | -0.55 | -3.48 | -5.77 |
