# Supplementary material for: SPServer: split-statistical potentials for the analysis of protein structures and protein–protein interactions
Source: BMC Bioinformatics. 2021 Jan 6;22:4. doi: 10.1186/s12859-020-03770-5 (PMC7788957; doi:10.1186/s12859-020-03770-5)
Supplement: Supplementary file 14 — Additional file 14. Table S2: Global scores of the native structure of Cysteine synthase A and two of its models. [file 12859_2020_3770_MOESM14_ESM.docx]

**Supplementary Table S2. Global scores of the native structure of Cysteine synthase A and two of its models.**

| **Fold** | **PAIR** | **ECOMB** | **ES3DC** | **ELOCAL** | **E3DC** | **E3D** | **ZPAIR** | **ZECOMB** | **ZES3DC** | **ZELOCAL** | **ZE3DC** |
| --- | --- | --- | --- | --- | --- | --- | --- | --- | --- | --- | --- |
| **Wildtype** | -7.816 | -123.7 | -9.015 | 3264 | -20.79 | -3358 | -2.364 | -4.147 | -1.330 | -3.785 | -2.005 |
| **Mutant** | -3.486 | -186.0 | -1.436 | 3213 | -21.65 | -3376 | -1.579 | -4.331 | -0.408 | -4.006 | -2.246 |
