# Supplementary material for: SPServer: split-statistical potentials for the analysis of protein structures and protein–protein interactions
Source: BMC Bioinformatics. 2021 Jan 6;22:4. doi: 10.1186/s12859-020-03770-5 (PMC7788957; doi:10.1186/s12859-020-03770-5)
Supplement: Supplementary file 15 — Additional file 15. Table S3: Comparison between global and quality metrics for the structures of CASP12 benchmark. [file 12859_2020_3770_MOESM15_ESM.docx]

**Supplementary Table S3: Comparison between global and quality metrics for the structures of CASP12 benchmark**.

|  | **ZES3DC** | | **ZPAIR** | | **PROSA** | | **DOPE** | | **GDT_TS** | | **TM score** | | **QCS** | |
| --- | --- | --- | --- | --- | --- | --- | --- | --- | --- | --- | --- | --- | --- | --- |
|  | **Mean** | **St. dev.** | **Mean** | **St. dev.** | **Mean** | **St. dev.** | **Mean** | **St. dev.** | **Mean** | **St. dev.** | **Mean** | **St. dev.** | **Mean** | **St. dev.** |
| **ZES3DC** | 1 | 0 | 0.78 | 0.02 | 0.72 | 0.03 | 0.68 | 0.05 | -0.44 | 0.05 | -0.51 | 0.05 | -0.49 | 0.05 |
| **ZPAIR** | 0.78 | 0.02 | 1 | 0 | 0.60 | 0.03 | 0.65 | 0.05 | -0.50 | 0.05 | -0.58 | 0.05 | -0.58 | 0.05 |
| **PROSA** | 0.72 | 0.03 | 0.60 | 0.03 | 1 | 0 | 0.69 | 0.05 | -0.43 | 0.05 | -0.47 | 0.04 | -0.41 | 0.05 |
| **DOPE** | 0.68 | 0.05 | 0.65 | 0.05 | 0.69 | 0.05 | 1 | 0 | -0.10 | 0.06 | -0.23 | 0.06 | -0.23 | 0.06 |
| **GDT_TS** | -0.44 | 0.05 | -0.50 | 0.05 | -0.43 | 0.05 | -0.10 | 0.06 | 1 | 0 | 0.97 | 0.01 | 0.83 | 0.04 |
| **TM score** | -0.51 | 0.05 | -0.58 | 0.05 | -0.47 | 0.04 | -0.23 | 0.06 | 0.97 | 0.01 | 1 | 0 | 0.85 | 0.04 |
| **QCS** | -0.49 | 0.05 | -0.58 | 0.05 | -0.41 | 0.05 | -0.23 | 0.06 | 0.83 | 0.04 | 0.85 | 0.04 | 1 | 0 |

**Legend Table S3: Mean Pearson correlation values and standard deviations of the comparison between the global scores of the SPServer (ZES3DC and ZPAIR), DOPE and PROSA (Pair Z-score) potentials, and TM, GDT_TS and QCS quality metrics for the structures of CASP12 dataset**. The correlation values are obtained by bootstrapping strategy using 1000 repetitions (see above). The Pearson correlation values between the scoring functions (using SPServer, DOPE and PROSA) and TM score, GDT_TS and QCS are negative because these metrics increase (from 0 to 1) with the quality of the structure, while scores are energy functions that decrease lower than 0 (being the lowest energy the best conformation).
