# Supplementary material for: SPServer: split-statistical potentials for the analysis of protein structures and protein–protein interactions
Source: BMC Bioinformatics. 2021 Jan 6;22:4. doi: 10.1186/s12859-020-03770-5 (PMC7788957; doi:10.1186/s12859-020-03770-5)
Supplement: Supplementary file 16 — Additional file 16. Table S4: Comparison of local (residue) profiles between SPServer and state-of-art methods DOPE and PROSA for the structures of CASP12 benchmark. [file 12859_2020_3770_MOESM16_ESM.docx]

**Supplementary Table S4: Comparison of local (residue) profiles between SPServer and state-of-art methods DOPE and PROSA for the structures of CASP12 benchmark.**

|  | **DOPE** | | **PROSA** | |
| --- | --- | --- | --- | --- |
|  | **Mean correlation** | **Standard deviation** | **Mean correlation** | **Standard deviation** |
| **ES3DC** | 0.18 | 0.14 | 0.21 | 0.14 |
| **PAIR** | 0.57 | 0.15 | 0.38 | 0.17 |

**Legend Table S4: Mean Pearson correlation values between the local (residue) scores of SPServer and state-of-art methods DOPE and PROSA for all structures of the CASP12 benchmark**. Each correlation value corresponds to the correlation of all the residue scores of a structure from the CASP12 benchmark.
